# Supplementary material for: Oxidation–reduction imaging of myoglobin reveals two-phase oxidation in the reperfused myocardium
Source: Basic Res Cardiol. 2024 Mar 18;119(3):435–51. doi: 10.1007/s00395-024-01040-6 (PMC11142982; doi:10.1007/s00395-024-01040-6)
Supplement: Supplementary file 1 — Supplementary file1 (PDF 862 KB) [file 395_2024_1040_MOESM1_ESM.pdf]

Supplemental Material

SUPPLEMENTAL METHODS:

Animal model and surgical procedures

C57BL/6 male mice aged 8-20 weeks and weighed 20-30g were bought from Charles River Laboratories, L'Arbresle, France. NMRI wt mice were bought to Janvier Laboratories, Saint Berthevin, France. NMRI MB knock-out (KO Mb) mice were provided by Prof Heiker at the Helmholtz Center in Munich, Germany. Mice were housed in groups of four in individually ventilated cages (NexGen - Allentown, USA – conventional animal facility) with standard nesting materials (cotton, tunnel) and *ad libitum* access to filtered water and standard diet (2018 global rodent diet, Envigo, France). The room temperature (housing and experiment) was maintained at 22°C ± 2°C) and the light cycle was at 12/12.

Animal procedures were performed following the guidelines from Directive 2010/63/EU on animal protection and have been approved by the institutional animal research ethical committee from Université Claude Bernard Lyon 1 and the French ministry (authorization: BH2012-65, APAFIS#10334-2017062221056351 and APAFIS #46204-2023120609212347). This study design was performed according to PREPARE guidelines, and experimentations were performed according to the ARRIVE guidelines. The number of animals required in this proof-of-technology study was determined *a priori* by power tests calculated with G*Power 3.1 software (one-way or two-way ANOVA with alpha: 0.05 / power 80% / effect size 0.5), which indicated a final inclusion of 5-6 animals per group.

Mice were randomized and four were operated each day. Lightsheet microscopy and image analysis were done blindly.

Mice were excluded when the clarification failed to reach enough transparency: inability to record the whole ventricles in lightsheet microscopy, imaging less than 2mm depth by confocal microscopy.

The mouse model of myocardial infarction was induced by occluding the left-anterior coronary artery, as previously reported by Harisseh R et al (2008). Mice were anesthetized by pentobarbital (73 mg/kg i.p.) and buprenorphine (0.075 mg/kg) as an analgesic subcutaneously. Animals were placed on a retro-regulated heating pad (target temperature: 37.5°C) on dorsal recumbency to be intubated and ventilated with a Physiosuite ventilator (Kent Scientific, USA). Mice underwent a left thoracotomy and an 8-0 polypropylene suture with a small curved needle was passed around the left anterior descending artery. Ischemia was confirmed by ST-segment elevation on the ECG and was maintained for 60 minutes. Ischemic postconditioning (IPoC) was achieved by three cycles of 1-minute ischemia and 1-minute reperfusion at the onset of reperfusion. Reperfusion was performed by releasing the suture, and animals were kept alive until sacrificed at different time points (15 minutes, 3 hours, and 24 hours). Before sacrifice, mice were re-operated, the suture was tightened, and Unisperse blue staining was injected to label the ischemic volume, named volume-at-risk (VAR). Upon sacrifice, transcortical perfusion with 10 ml ice-cold phosphate-buffered saline (PBS) at a 2ml/min rate was done in mice to wash out blood from the heart. Following PBS, 5 ml of 4% paraformaldehyde (PFA) was perfused for fixation. Hearts were then dissected. Sham-operated animals were used as control, where the suture was passed and not tied. Animals were randomly assigned to each group throughout the study.

Troponin I measurement

After collection, blood samples were centrifuged at 500G for 5 minutes to collect serum. Serum samples were stored at -80°C and thawed only once for the high sensitivity Troponin I assay at the Hospital laboratory.

Myoglobin/myocardium oxidation/reduction

The preparation has been derived from Whitburn et al.. Pure horse myoglobin (SIGMA, ref: M1882; Saint Louis, MO) was dissolved at 0.2g/ml in PBS pH 7.2 (solution A). Dithionite, $Na_2S_2O_4$ (SIGMA, ref: 1065051000; Germany) was prepared at 100mM in PBS pH 7.2 (solution B). Solution enriched in CarbMyoglobin (CarbMb) was prepared by mixing: 500µl solution A + 494µl PBS + 60µl solution B ($Na_2S_2O_4$ 6mM final). Solution enriched in MetMyoglobin (MetMb) was prepared by adding H_2O_2 0.03, 0.3, or 3% (V/V) to solution B. These solutions are a mixture of Carb, Met, and Ferryl-Mb in different proportions. For instance, MetMb is present in the CarbMb solution due to the auto-oxidation mechanism, while Ferryl-Mb could be produced in excess of H_2O_2. Although these mixtures would have been inappropriate for the specific spectral characterization of each species, they were a good model for the myocardium. In addition, they demonstrated that spectral differences between solutions enriched with either CarbMb or MetMb could be detected even in mixed myoglobin populations.

Post ischemia-reperfusion and cardioprotection procedures, myocardium were cut to small pieces before their incubation in either PBS, H_2O_2 0.03% (V/V) to induce oxidation or in 6mM NaS_2O_4 to induce reduction. Half of the myocardium pieces were then cleared in X-clarity and finally imaged by spectral fluorescence, while the other halves were imaged directly.

Heart active clarification.

Dissected hearts were placed in 4% PFA for 24 hours minimum, protected from light in the fridge at 4°C in a 1.5ml Eppendorf tube. Hearts were then incubated in a polymerization solution constituting PBS 1X, acrylamide 4%, and VA-044 0.25% for 24 hrs at 4 ^{o}C in 5ml Eppendorf tubes. To polymerize the gel, hearts were incubated at 37 ^{o}C for 3 hours under gentle agitation in a dark box to prevent myoglobin photooxidation. After a brief wash with PBS, hearts were placed in the X-clarity machine chamber for active clarification via the Logos® electrophoretic tissue-clearing solution. Hearts were cleared in 24 hours at 1.3A and 30rpm.

Isolation of Adult cardiomyocytes

Following the protocol adapted from O'Connell et al. (2007), the mouse heart was perfused by Langerdorff with perfusion buffer for 3 minutes and then with digestion buffer for 8 minutes at 37°C. Once the digestion was complete, the heart was placed in a 100-mm dish and cut into small pieces in the digestion buffer. A mechanical dissociation was done by gentle pipetting. Next, the cell suspension was centrifuged at x20g at room temperature for 3 min. The pellet was resuspended in a 10 mL stop buffer containing 200 mM of ATP. A calcium increase in the buffer was realized in several successive steps interspersed by centrifugations (3 min, x20g) until a final calcium concentration of 0.9 mM. Then, isolated cardiomyocytes were plated on a glass slide and ready for immunostaining.

Immunofluorescence

After isolation, the cardiomyocytes were fixed in 2% PFA for 15 minutes at RT, followed by 40 minutes of permeabilization (PBS 5% milk 0.1% triton X100+0.5% saponin) at RT. Cells were then incubated in the blocking solution by adding 5% dry milk in PBS for 1 hour. Primary

antibodies (mouse monoclonal anti-GRIM-19, Santa Cruz, SC-514111, 1/200 & rabbit monoclonal anti-myoglobin, R&D systems, MAB97203,1/100) were incubated overnight at 4°C. Cardiomyocytes were then washed in PBS three times for 5 minutes. Secondary antibodies from Thermo Fisher Scientific (AF488 anti-mouse, A11034,1/1000 & AF647 anti-rabbit, AF21244, 1/1000) were incubated at RT for 2 hours, followed by three washes for 5 minutes each. Stained cardiomyocytes were imaged by confocal microscopy according to the fluorescence properties of the fluorochromes.

Confocal imaging

Cardiomyocyte imaging was done on a Nikon Eclipse Ti, A1R confocal microscope with a 40x oil objective. Bandpass filters were used to isolate the green and red fluorescence emissions (laser at 488nm and 633nm).

Before imaging, clarified hearts were placed in a refractive index mounting solution (RIMS) and X-ClarityTM Mounting solution (Logos Biosystems) for at least 2 hours. Hearts or pieces of myocardium were then placed in a container with RIMS for confocal imaging. A 10x objective was used for all the acquired images. Mosaic Z-stack spectral images of 100 µm thickness were acquired via the excitation of all the lasers (diode 405 nm (blue), argon ion 488-514 nm (blue-green), diode 561 nm (orange-red) and diode 642 nm (deep red). Images were acquired by averaging 4-scanning lines for noise reduction and better resolution. Spectral detection was done using a 32 channels GASP on a bandpass 400 to 750nm at maximum.

Identical settings were used for the spectroscopy of pure horse myoglobin in solution. A specific control for glass slide reflection was utilized to correct the leak around the dichroic mirrors (**Supplemental Figure S2A**).

Single-plane illumination microscopy

An ultramicroscope (LaVision BioTec) was used to perform imaging. The light sheet was generated by a 633 nm laser (Coherent Sapphire Laser, LaVision BioTec) and two cylindrical lenses. A binocular stereomicroscope (MXV10, Olympus) with a 2x objective (MVPLAPO, Olympus) was used at 6.3x. Samples were placed in an imaging reservoir of 100% quartz (LaVision BioTec) filled with X-ClarityTM Mounting solution (Logos Biosystems). A Andor Neo SCMOS camera (2560 x 2160-pixel size, LaVision BioTec) was used to acquire images. Hearts were pre-incubated in X-Clarity^{TM} Mounting solution and protected from light in a fridge at 4°C (Logos Biosystems) overnight.

Magnetic resonance imaging (MRI) setup

Twenty-four hours ischemia-reperfused mice were placed in an anesthesia induction box with a mixture of 4% isoflurane gas and air containing 30% oxygen delivered at a rate of 1.5/min. Animals were then placed supine in a homemade dedicated plastic bed and maintained during the entire MRI protocol under anesthesia at 1.5 to 2.5% isoflurane (rate 0.6/min) delivered via a cone mask. Body temperature was maintained at 36.5°C using thermo-regulated water via a circuit integrated within the dedicated bed.

ECG-based cardiac and respiratory gating was carefully obtained via a triggering unit (ECG Trigger Unit HR V2.0, Rapid Biomedical, Würzburg, Germany). Front paws were wrapped in copper foil electrodes, and the peripheral ECG signal was derived via silver wire. The ECG signal was detected by equipped low-pass and high-pass filters to eliminate interference with gradient and Radio Frequency pulses and low-frequency distortion as much as possible. A breathing sensor

was also placed on the abdomen for respiratory gating to continuously monitor the respiration rate measured using the same specialized device.
MRI acquisitions were performed in vivo on a horizontal 7T BRUKER BioSpec MRI system (Bruker Biospin MRI GbmH Bruker, Germany) equipped with a set of gradients of 440 mT/m and controlled via Bruker ParaVision 5.1 workstation. A Bruker birdcage volume coil (outer diameter = 112 mm and inner diameter = 72 mm) was used for the signal transmission, and a Bruker single loop surface coil (15 mm diameter) was used for signal reception, being positioned on the animal thorax to target the heart.
After a quick localizer, short axis views ("two chambers") are obtained on a slice plane going through the middle of the mitral valve to the apex. Finally, long axis views ("four chambers") are obtained on a slice plane through the apex of the heart and perpendicular to the inter-ventricular septum.
In order to quantify the myocardial infarction volume using MRI, Late Gadolinium Enhancement (LGE) technique was employed 10 minutes after retro-orbital injection of 150µL Gadolinium-DOTA of 0.2 mM/mL. Bidimensional T1 weighted gradient echo image with the following parameters: echo time (TE) = 2. 8ms, repetition time (TE) = 116.1ms, flip angle (FA) = 70 deg, number of averages = 4. A total of 15 slices of 700µm slice thickness were obtained in short axis orientation within a field of view (FOV) of 4 x 4 cm^2, and a matrix size of 256 x 256 pixels, providing an in-plane resolution of 156 x 156 µm, for an acquisition time of 1'29". Due to the ECG/respiration triggering, the actual acquisition time was about 7 minutes.

Image analysis
Spectral images taken with Nikon A1R plus confocal microscope were analyzed with NIS-Elements software (Nikon). Linear unmixing was used to deconvolute spectral signals.
Semi-automatic segmentation of 1) the volume-at-risk and 2) the oxidized volume (LSM images and MRI images) as well as of the healthy left ventricle volume was performed using AMIRA-AVIZO visualization and analysis software (ThermoFisher Scientific, Oregon, USA). Extraction of the gradient of fluorescence was performed with a custom-made Python script for AMIRA-AVIZO; this script integrated within an AMIRA module the region of interest (ROI) selections, computations —either mean profile along the ROI main axis or first derivative of the mean profile (gradient)— and file export.
Statistical analysis of pixel distribution by fluorescence intensity has been performed with MATLAB 2014a (The MathWorks, Natick, MA, USA) and Image Analyst toolbox (Image Analyst (2021). Fit Multiple Gaussians (https://www.mathworks.com/matlabcentral/fileexchange/74408-fit- multiple-gaussian), MATLAB Central File Exchange) was used for the gaussian fit.

Data analysis
Each group was achieved with 6 hearts, and at least 5 of them were finally included at the end of the process. Values are expressed as median ± CI 95%. The data were analyzed, and graphs were plotted using GraphPad Prism Software (SanDiego, USA) for statistical analysis. Mean values were compared using either an unpaired t test with Welch's corrected test (2 groups) or Brown-Forsythe and Welch ANOVA test with Dunnett multiple comparison post-test (≥3 groups). Statistical significance was denoted by (*) for $P<0.05$.

SUPPLEMENTAL FIGURES:

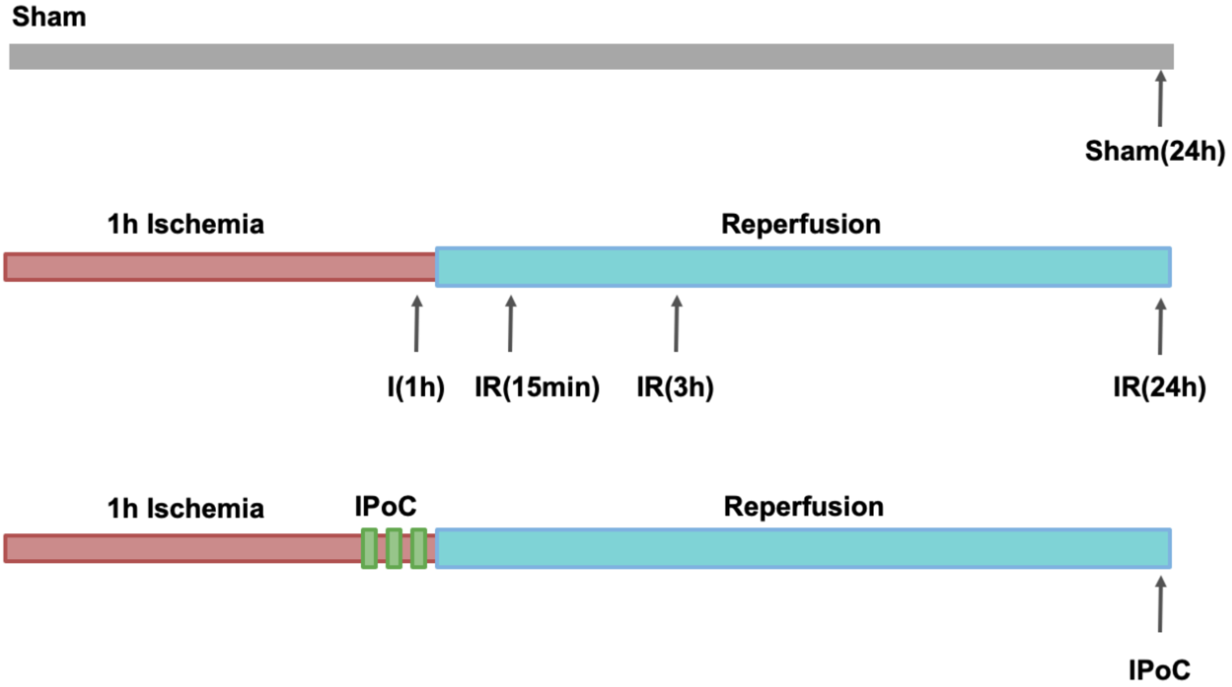

FIGURE S1. Experimental protocols of myocardial infarction. Schemes represent the timing of the different experimental groups: sham, ischemia: I(duration), ischemia-reperfusion: IR(duration), and ischemia-reperfusion + ischemic post-conditioning (IR(24h)+IPoC).

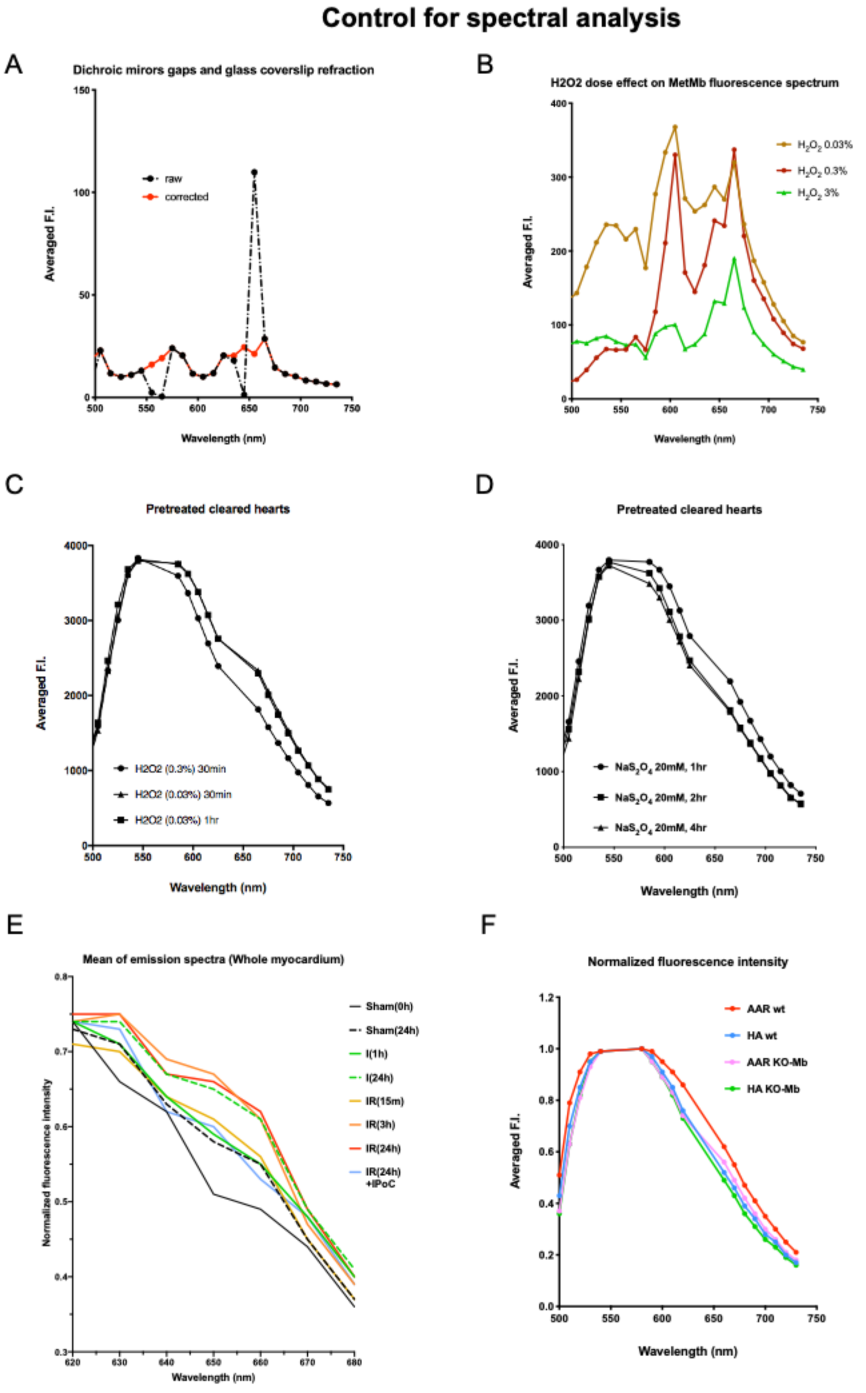

Fig S2

FIGURE S2. Additional controls and information about spectral analysis of myoglobin fluorescence and endogenous fluorescent signal in cleared myocardium. **A.** Spectrum of fluorescence recording with refractive solution (Logos Biosystems) alone show glass-induced light diffraction and leak through the 633 dichroic mirror (black line), after correction of the leak around the dichroic mirror is done by removal of the adjacent channel on the spectral detector (red line). **B.** Fluorescence spectra of pure horse myoglobin measured with the confocal microscope under

illumination with laser lines: 405, 488, 561, and 633nm and detection by a spectral avalanche photodiode. Myoglobin was enriched in oxidized form (MetMb) by treatment with different concentrations of hydrogen peroxide (H_2O_2). **C.** Fluorescence spectra of heart treated with different concentrations of hydrogen peroxide (H_2O_2) before clarification. **D.** Fluorescence spectra of heart treated with 20mM dithionite (NaS_2O_4) for a different duration before clarification. **E.** Example of endogenous fluorescence spectra in the red wavelength range extracted from one area-at-risk per experimental group. **F.** Averaged normalized intensity fluorescence spectra from heart optical slices of 3 wild type (wt) and 3 myoglobin knock-out (KO-Mb) mouse hearts in both healthy area (HA) and area-at-risk (AAR).

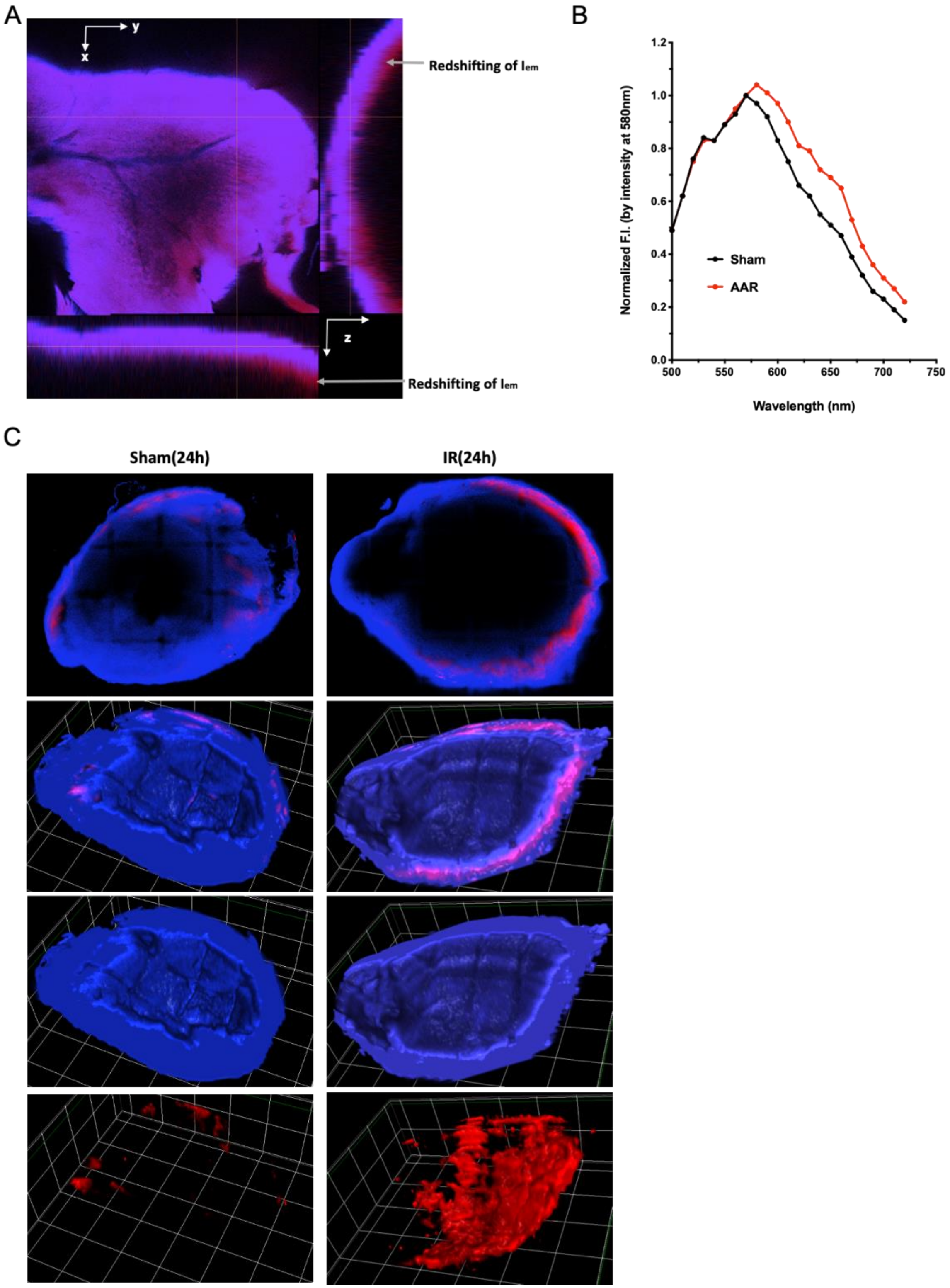

FIGURE S3. ***Control for red shift in depth and linear unmixing of AAR and sham spectra***. **A.** Spectral imaging with a confocal microscope and orthogonal project of the image stack reports the absorption of blue photons within the inner margin of the myocardium. **B.** Fluorescence spectra of

voxels from a sham heart and for the area-at-risk of a 24h-reperfused heart. **C.** Image segmentation of a sham (left column) and a 24h-reperfused heart (right column) by linear unmixing of the mean spectra: sham and AAR presented in B. From top to bottom: confocal slice, 3D rendering volume with healthy volume (blue) and volume-at-risk (red), healthy volume alone and volume-at-risk.

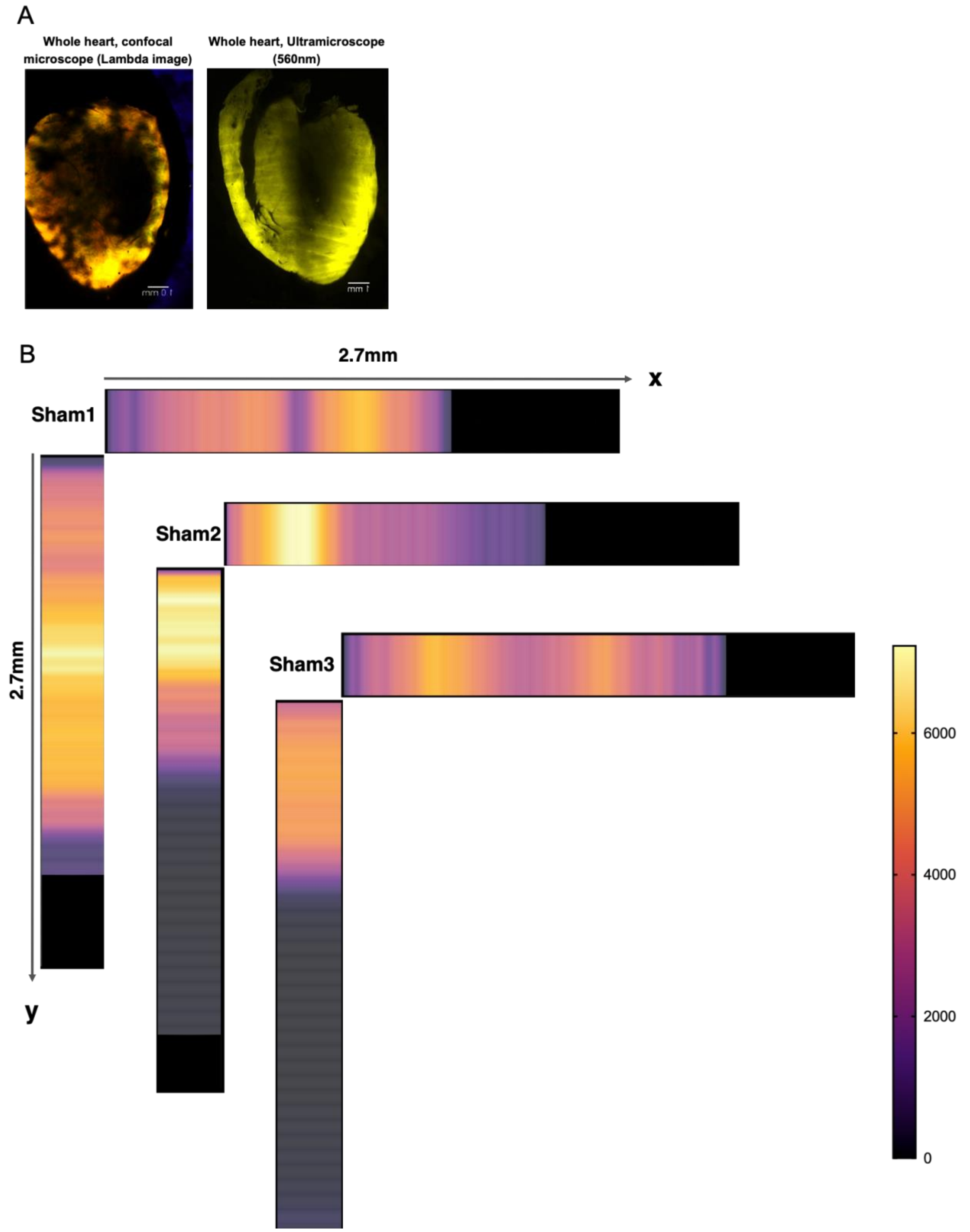

FIGURE S4. Fluorescence heterogeneity along the light path. A. comparison of a sagittal slice extracted from a 3D reconstruction of images taken by confocal microscopy (left) or light sheet

microscopy (right) of the same heart. **B.** Fluorescence intensity along x and y axis of the light sheet from 3 different sham hearts shows great heterogeneity in light absorption.

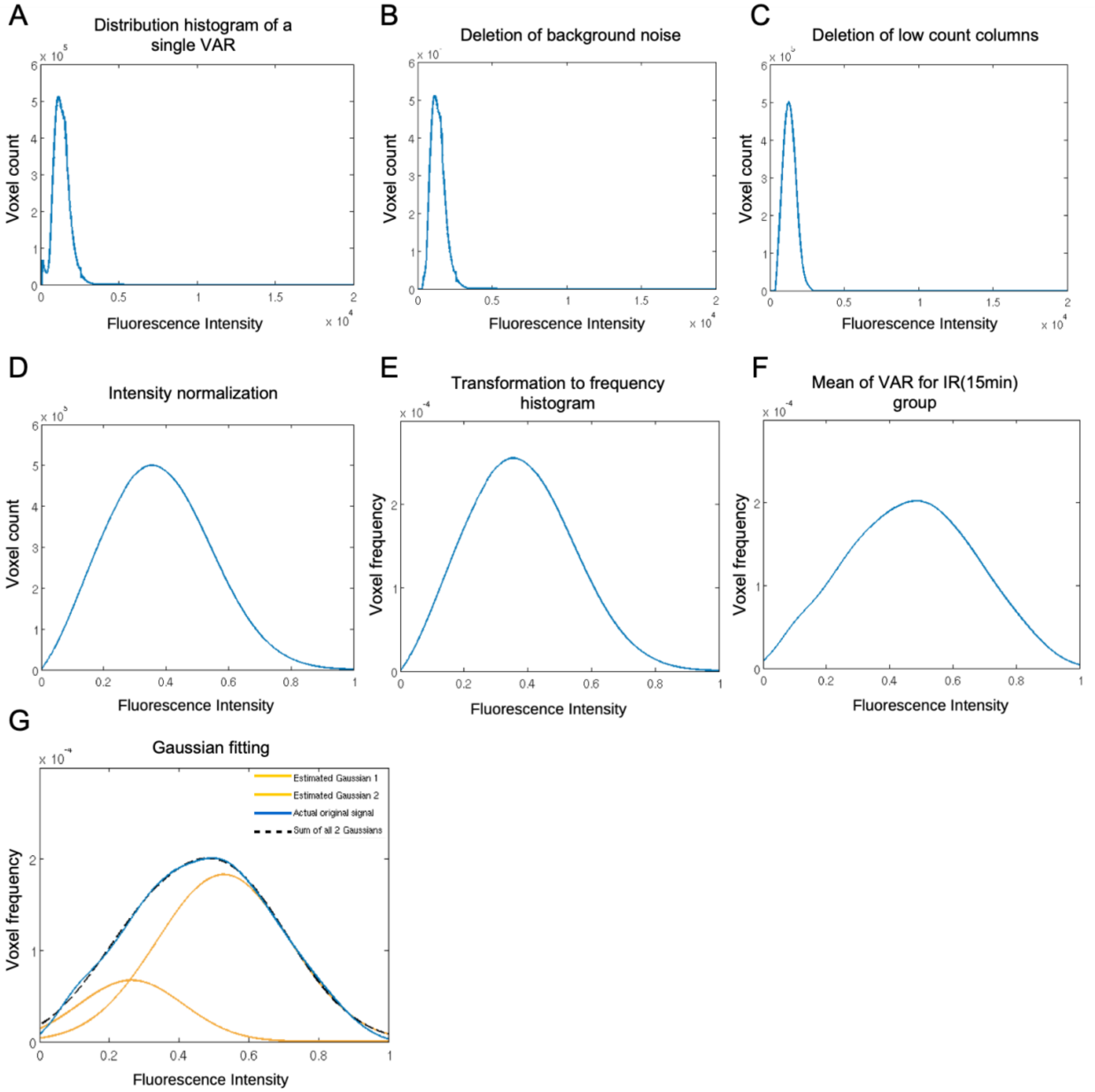

FIGURE S5. Pipeline for normalization of distribution histograms of voxels per fluorescence intensity. A. Voxels from one volume-at-risk were plotted on a distribution histogram. In order to normalize experimental noise (as previously described): 1) low intensities (background noise) was deleted (**B**), 2) low-frequency intensities at the foot of the distribution were removed at a fix threshold of 0.002% (**C**), 3) values of intensity were normalized between 0 and 1 (where 0 is the first intensity value with more than 1 point and 1, the last intensity value with more than 1 point; **D**). For each histogram, the total number of voxels was normalized before each column was

divided by the total number of voxels to create the frequency histogram. **E.** A Gaussian function was fitted on each frequency histogram. **F.** Frequency histograms generated from hearts of the same experimental group were averaged. **G.** Image Analyst toolbox (Image Analyst (2021). Fit Multiple Gaussians (https://www.mathworks.com/matlabcentral/fileexchange/74408-fit-multiple-gaussian), MATLAB Central File Exchange) was used for the gaussian fit **(G)**. Number of gaussian and initial positions were needed for gaussian fitting.
